# A case series of 99mTc-MDP bone scintigraphy (planar and SPECT CT) in mucormycosis in the era of COVID 19

**DOI:** 10.3389/fradi.2025.1683149

**Published:** 2025-11-20

**Authors:** Vandana Kumar Dhingra, K. Vidhya, Amit Kumar, Amit Kumar Tyagi

**Affiliations:** 1 Department of Nuclear Medicine, AIIMS Rishikesh, Rishikesh, India; 2 Department of ENT, AIIMS Rishikesh, Rishikesh, India

**Keywords:** bone scintigraphy, COVID-19, mucormycosis, SPECT/CT, head and neck

## Abstract

Mucormycosis is a serious fungal infection affecting immunocompromised individuals, caused by fungi from the Mucorales order, particularly Rhizopus species. It primarily spreads through inhalation of spores, with diabetes, cancers, organ transplants, immunosuppressive drugs, and COVID-19 being major risk factors. The infection manifests in various forms such as encephalic, cutaneous, gastrointestinal, pulmonary, and rhino cerebral, often leading to tissue necrosis and blood vessel invasion. Imaging diagnosis is aided by CT and MRI scans, while 99m Tc MDP bone scintigraphy has found to be a more accurate imaging tool to look for bone remodelling and erosive changes associated with invasive fungal sinusitis including mucormycosis. Treatment involves prompt surgical debridement and addressing the underlying immune deficiency. Here we present a series of cases where 99m Tc MDP bone scintigraphy played a key role in management of mucormycosis of the head. In conclusion, 99mTc MDP scintigraphy is a promising tool for evaluation, guiding diagnosis and management of mucormycosis.

## 1 Introduction

Mucormycosis is a severe opportunistic fungal infection caused by fungi from the Mucorales order, most commonly Rhizopus species, found in soil and decaying organic matter (1, 2). The infection primarily occurs through inhalation of spores that settle in the lungs and paranasal sinuses, although it can also result from direct skin inoculation or ingestion. Individuals with compromised immune systems are at high risk, particularly those with diabetes mellitus, cancers such as leukemias and lymphomas, organ transplants, immunosuppressive medications, and conditions like COVID-19, which weaken the immune system (3–5). Mucormycosis can manifest in several clinical forms, including encephalic (brain), cutaneous (skin), gastrointestinal, pulmonary (lung), and rhinocerebral (nose and brain) types. The infection is characterized by tissue necrosis, blood vessel invasion, and thrombosis, progressing rapidly and often requiring urgent medical attention. The most prevalent form, rhinocerebral mucormycosis, has a high mortality rate, particularly if the patient's immune system cannot be restored (6). Early diagnosis is crucial and typically involves imaging techniques like CT and MRI scans, which help assess the extent of anatomical involvement, though they are not definitive on their own (2, 7). Bone scan has been found to be a more accurate method to image bone remodelling and erosive changes associated with invasive fungal sinusitis including mucormycosis. Effective treatment necessitates early and aggressive surgical debridement to remove necrotic tissue, alongside medical management to strengthen the immune system and address underlying conditions. In the context of COVID-19, increased awareness among healthcare providers is essential for early detection and prompt intervention to improve patient outcomes (8). Post COVID we received multiple cases of mucormycosis where rapid diagnosis, imaging and surgery were performed. Accuracy of detection of disease involvement and diagnosis of recurrence were of prime importance as they would help guide surgical extent. In many cases bone scintigraphy was useful, we discuss a few examples from these cases.

## 2 Materials and methods

### 2.1 Study design

This was a prospective study conducted over a 3-month period during the COVID-19 era, in which patients were selected based on clinical symptomatology, prior immunocompromised status, pathological examination, and anatomical imaging findings. All patients referred from the ENT department were included to minimize selection bias.

### 2.2 Patient selection

#### 2.2.1 Inclusion criteria

- Patients were selected based on clinical suspicion of post-COVID fungal mucormycosis.
- Both preoperative and postoperative cases were included to assess disease extent and evaluate postoperative residual or recurrent activity.
- Clinical indications for imaging included facial or orbital pain, swelling, nasal congestion, black eschar, loosening of teeth, and non-healing ulcers.
- Diagnosis classification followed the EORTC/MSG (European Organization for Research and Treatment of Cancer/Mycoses Study Group) criteria, integrating- Clinical presentation Radiological findings (CT/MRI) Microbiological or histopathological confirmation (wherever available).
- Only patients with a confirmed history of COVID-19 infection were included, and the time interval since infection was documented to establish temporal association with mucormycosis onset.
- For each anatomical site, the reference standard for disease involvement was defined as: Histopathological confirmation, wherever available.
- Intraoperative findings, when histology was not feasible.
- Composite clinical reference standard adjudicated by a multidisciplinary team (MDT), incorporating clinical, radiological, and therapeutic response parameters.
- When histopathology was unavailable, true site-specific involvement was determined based on concordant radiological findings and intraoperative or clinical evidence validated through MDT review.

#### 2.2.2 Exclusion criteria

- Patients without a documented history of COVID-19 infection were excluded.
- Cases with fungal sinusitis due to other etiologies (e.g., aspergillosis or candidiasis) confirmed by microbiology or histopathology were excluded.
- Patients with incomplete clinical records, inadequate imaging data, or lack of confirmatory evaluation were excluded to maintain diagnostic accuracy.
- Subjects with poor-quality or artifact-laden SPECT/CT or bone scintigraphy images were excluded.
- Patients with prior unrelated maxillofacial or orbital surgeries, lost to follow-up, or lacking postoperative evaluation were excluded.
- Cases where site-specific disease confirmation could not be established via histopathology, intraoperative findings, or MDT adjudication were omitted from analysis.

#### 2.2.3 Patient preparation

- Patients were instructed to remain well-hydrated and drink approximately 1 L of water between radiotracer injection and imaging.
- Fasting was not required before the 99mTc-MDP bone scan.
- Patients were made to lie still during image acquisition to minimize motion artifacts.

#### 2.2.4 Instrumentation

- 99Mo-99mTc Molybdenum-Technetium generator.
- Cold radiopharmaceutical kit for MDP injection.
- Dual-head gamma camera GE NMCT 670 (integrated SPECT/CT by GE).
- GE Xeleris workstation.

#### 2.2.5 Radiopharmaceutical preparation and injection procedure

- Radiopharmaceutical was tagged and allowed to stand for 10 min.
- Quality control was performed using thin-layer chromatography and found to be >99%.
- 15–20 mCi of 99mTc-MDP was injected intravenously.
- Imaging was performed at 3 h post-injection.

#### 2.2.6 Imaging procedure

##### 2.2.6.1 Planar imaging

- Delayed whole-body images were acquired at 180 min post-injection in anterior and posterior views.
- Gamma camera moved at 13 cm/min with a 256 × 1,024 matrix, zoom factor 1, pixel size 2.4.
- Spot views were obtained using 256 × 256 matrix, zoom factor 1, for 2–5 min.

##### 2.2.6.2 SPECT/CT acquisition

- SPECT acquisition: 128 × 128 matrix, 128 projections over 360°, 20–25 s per projection.
- Low-dose CT: 80 mA, 120 kV, reconstructed in axial, sagittal, and coronal planes.

##### 2.2.6.3 Image reconstruction and motion control

- Dual-head gamma camera equipped with LEHR collimator.
- Patients instructed to minimize movement; head immobilization using straps/cushions when required.
- Sedation not routinely administered.
- Metallic dental artifacts managed via patient positioning (open-mouth/lateralized head) and metal artifact reduction algorithms.
- SPECT reconstructed using OSEM algorithm (8 iterations, 10 subsets).
- Attenuation correction from low-dose CT and scatter correction applied.
- Post-processing Butterworth filter: cut-off 0.5 cycles/cm, order 10.
- Two blinded reviewers independently reviewed and reported all scans.

#### 2.2.7 Confounders and bias

Potential confounders included post-operative changes, chronic osteitis/osteomyelitis, and dental pathology. Differentiation between active fungal osteomyelitis and post-surgical remodeling was achieved using clinical correlation, temporal imaging changes, and CT/MRI anatomy.

- Homogeneous low-grade uptake confined to surgical margins without progression → interpreted as post-operative remodeling.
- Focal/asymmetric uptake beyond surgical margins with cortical breach or soft tissue extension → interpreted as active disease.
- Dental pathology excluded via orthopantomogram, dental examination, and localized uptake patterns.

For ambiguous cases (e.g., Cases 5–6), clinical response to antifungal therapy or debridement and follow-up scans were used to confirm specificity. Inflammatory markers (CRP, ESR) and timing of antifungal therapy were also considered to contextualize tracer uptake.

#### 2.2.8 Image interpretation criteria

- Bone lesions with increased 99mTc-MDP uptake were considered positive for bony involvement in mucormycosis.
- SPECT/CT findings were reviewed with surgical and infectious disease teams to document impact on management:
  ○ Additional surgical debridement
  ○ Expansion of surgical field
  ○ Targeted biopsy guidance
- Operative correlation recorded per anatomical site where available.

## 3 Case descriptions

### 3.1 Case 1

**Figure 1:**
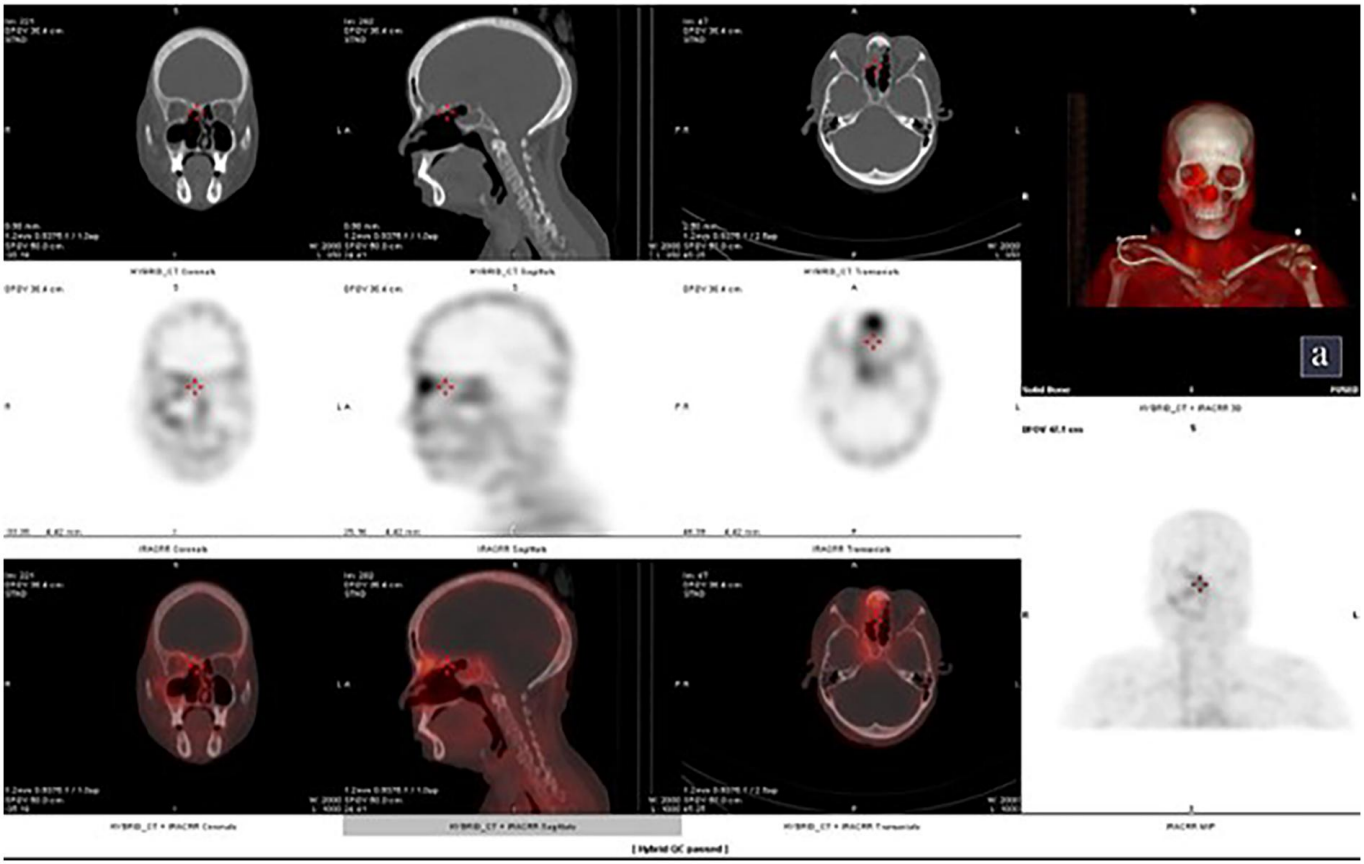
Case 1: additional SPECT/CT images showing involvement of right frontal bone, nasal septum, lesser wing of sphenoid on the right side, and body of sphenoid.

A 59-year-old female, with Type 2 Diabetes mellitus and hypertension since 20 years, presented with recurrent facial pain since 2 months. She had a recent history of sinonasal surgery for mucormycosis. She had no history of fracture, dental extraction, back pain, or any other joint pain. Laboratory parameters were significant for raised Alkaline phosphatase of 407 IU/L and normal serum calcium. KOH mount was found to be negative for fungus. MRI was suggestive of post-operative changes with residual disease in right maxillary sinus, ethmoidal cells, frontal and sphenoid sinus with right orbital and meningeal involvement. 99m Tc MDP Bone Scan: planar images (Supplementary Figure a) revealed involvement of right nasal and maxillary region, whereas additional SPECT/CT images (Figure 1) revealed involvement of frontal bone (right side), nasal septum, lesser wing of sphenoid on the right side, and body of sphenoid.

Incremental value of 99m Tc MDP Bone scintigraphy:

a. Bone scintigraphy (SPECT/CT) revealed additional involvement suggesting active residual disease in the frontal bone, nasal septum, lesser wing and body of sphenoid.
b. These findings extended beyond what MRI suggested and helped delineate skull base involvement, refining the disease extent.

### 3.2 Case 2

**Figure 2:**
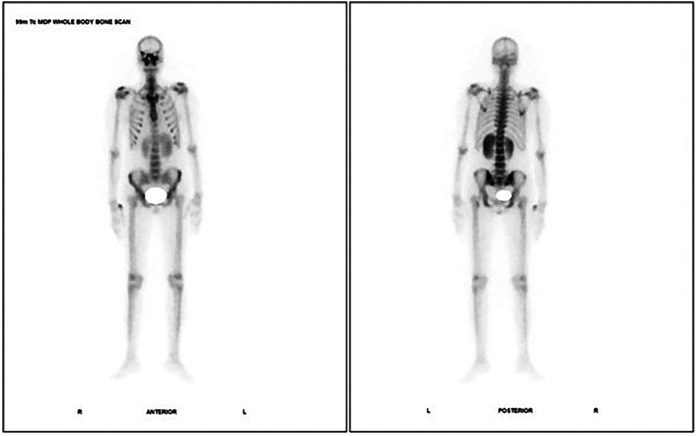
Case 2: 99m Tc MDP bone scintigraphy- planar images showing nasal (left > right) and maxillary region.

A 42 year old female, recently diagnosed with Type 2 Diabetes mellitus, post COVID-19 presented with headache associated with swelling on right side of face and eye since 2 months and was diagnosed with mucormycosis for which she underwent a bilateral marginal mandibulectomy with bilateral frontal, ethmoidal and sphenoidectomy 4 weeks back. Plain CT Head was suggestive of residual mucosal thickening with bony erosions and post-operative changes. Contrast MRI (CEMRI) was suggestive of pachymeningitis with orbital apex involvement. 99m Tc MDP Bone scintigraphy: Planar images (Figure 2) revealed nasal (left > right) and maxillary region, whereas additional SPECT/CT images additionally revealed involvement of cribriform plate and bilateral greater wing of sphenoid.

Incremental value of 99m Tc MDP Bone scintigraphy:

a. SPECT/CT detected additional cribriform plate and bilateral greater wing of sphenoid involvement.
b. This extended active residual disease beyond maxillary sinus (on MRI), emphasizing bilateral skull base infiltration was not clearly visualized on MRI.

### 3.3 Case 3

**Figure 3:**
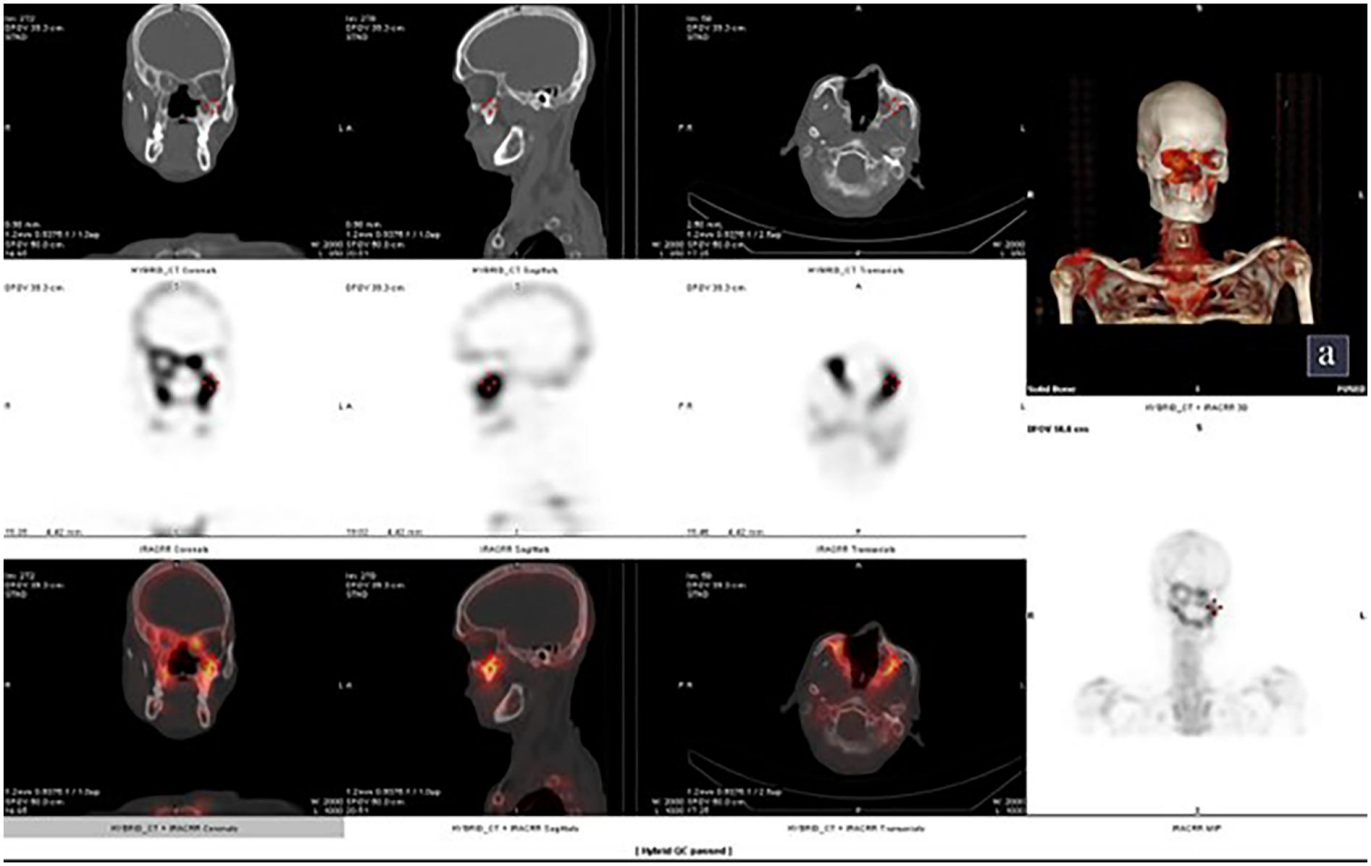
Case 3: additional SPECT/CT images showing increased tracer uptake in the lesser wing of sphenoid right side, floor and orbital plate of right maxilla, right pterygoid plate, right zygomatic bone, right mastoid bone with clouding of right mastoid air cells.

A 51 year old male, recently diagnosed Type 2 Diabetes mellitus and hypertension (not on regular medications), presented with swelling and decreased vision in right eye since 8 days, jaw pain since 8 days and decrease vision of left eye since 4 days, eventually leading to his diagnosis of mucormycosis for which he underwent a bilateral Medial maxillectomy with right orbital exenteration 2 weeks back. He had a history of COVID 19 1 month back. He had no history of fracture, dental extraction, back pain, or any other joint pain. KOH Mount was suggestive of mucormycosis. CEMRI was suggestive of bilateral maxillary ethmoid and sphenoid flow intensities suggestive of sinusitis and also superior part of extrachoanal space of right orbit was also involved. 99m Tc MDP Bone scintigraphy- planar images (Supplementary Figure b) revealed right maxillary region involvement with SPECT/CT images (Figure 3) revealing additional sites of involvement in lesser wing of sphenoid bilaterally, ethmoidal air cells, orbital walls of right orbit, nasal bone and flow of bilateral maxilla.

Incremental value of 99m Tc MDP Bone scintigraphy:

a. Planar imaging showed only right maxilla; SPECT/CT revealed bilateral lesser wings of sphenoid, ethmoidal air cells, orbital walls, nasal bone, and bilateral maxilla.
b. Allowed better evaluation of active residual/recurrent disease of orbital and ethmoidal spread, important for further surgical evaluation or radiotherapy.

### 3.4 Case 4

A 18 year old male, known case of type 1 diabetes mellitus since on insulin, presented with history of throat pain, numbness and swelling over right eye, eventually leading to his diagnosis of mucormycosis for which he underwent a partial maxillectomy of right suprastructure and incision and drainage of abscess of right eyelid and right side of neck few weeks back. He had no history of COVID 19 pneumonia. CEMRI Brain was suggestive of mucosal thickening in bilateral maxillary ethmoidal, right sphenoid and right frontal sinus with right cavernous sinus and Internal Carotid Artery thrombosis. KOH MOUNT was suggestive of negative for fungal elements. Histopathology: positive for invasive mucormycosis. 99m Tc MDP Bone scintigraphy: Planar images (Supplementary Figure c) revealed lesion involving right nasal and right maxillary region and additional SPECT/CT images revealed increased tracer uptake in the lesser wing of sphenoid right side, floor and orbital plate of right maxilla, right pterygoid plate, right zygomatic bone, right mastoid bone with clouding of right mastoid air cells.

Incremental value of 99m Tc MDP Bone scintigraphy:

a. SPECT/CT showed uptake in right zygomatic bone, pterygoid plate, mastoid bone, and floor/orbital plate of maxilla.
b. Provided posterior skull base and temporal bone involvement, not seen on MRI, guiding surgical planning in this patient and leading to more aggressive management.

### 3.5 Case 5

**Figure 4:**
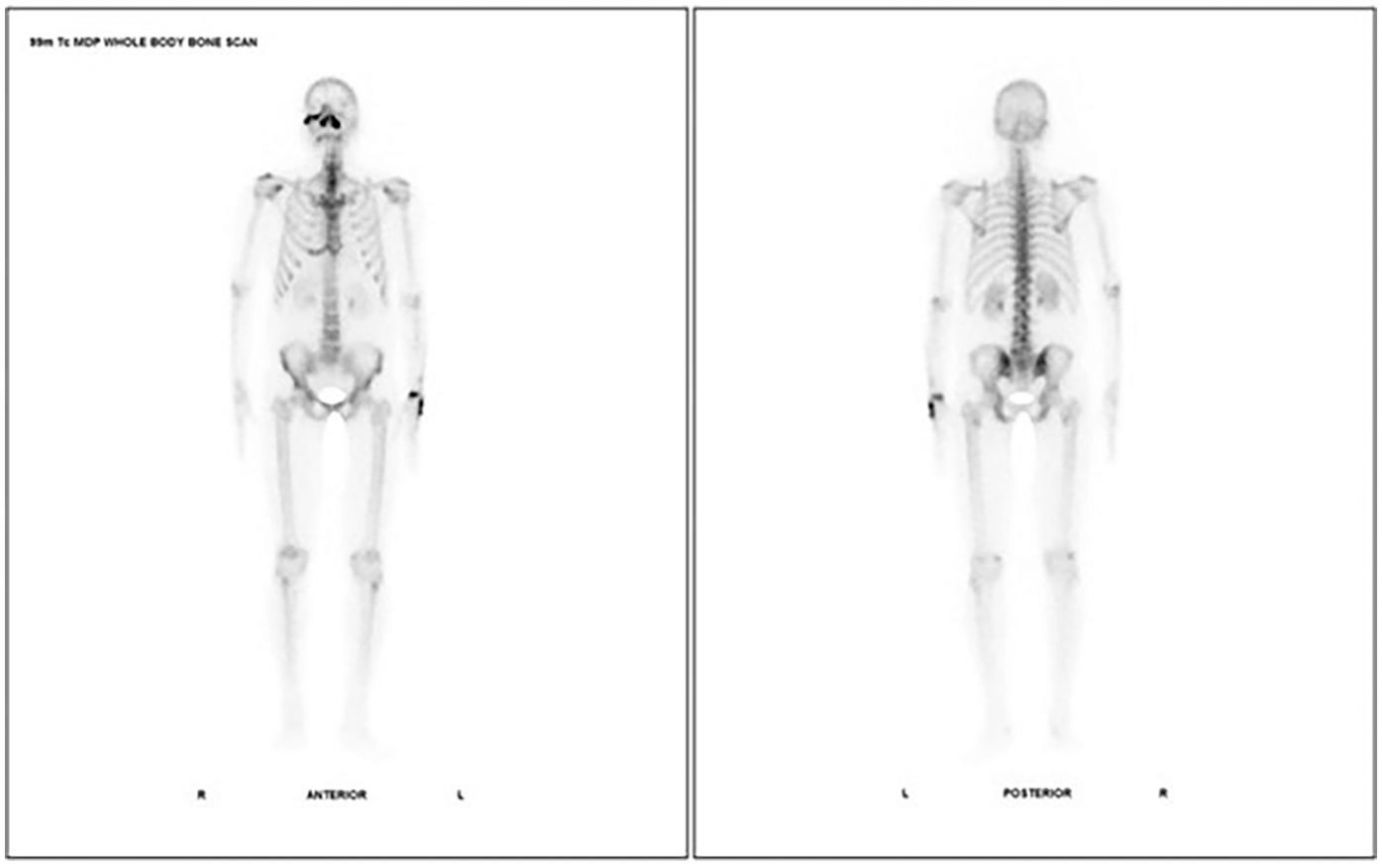
Case 5: 99m Tc MDP bone scintigraphy- planar images showing involvement of nasal and right maxillary region.

A 49 year old male, known Type 2 diabetes mellitus presented with right sided facial palsy and headache since 1 day, swelling and discoloration on right side of face since 4 days which led to diagnosis of mucormycosis for which he underwent a right inferior partial maxillectomy. She had no history of fracture, dental extraction, back pain, or any other joint pain. Plain CT head was suggestive of residual mucosal thickening in bilateral maxillectomy in right frontal and ethmoidal sinuses with post op changes. KOH mount pre-operative was negative for mucor and post operatively was found to be suggestive of mucor mycosis. 99m Tc MDP Bone scintigraphy- Planar images (Figure 4) revealed involvement of nasal and right maxillary region with additional SPECT/CT images revealing increased tracer uptake in the right side of lesser wing of sphenoid, greater wing of sphenoid right side, right ethmoidal air cells with mucosal thickening, anterior and medial wall of right maxilla, floor and medial wall of left maxilla, right pterygoid plates, temporal process of right zygomatic bone and chondral part of right 6th rib along with another suspicious osteolytic lesion with mildly increased tracer uptake in C2 vertebra.

Incremental value of 99m Tc MDP Bone scintigraphy:

a. SPECT/CT revealed multiple new sites: right greater/lesser wing of sphenoid, ethmoidal air cells, walls of both maxillae, right pterygoid plates, zygomatic bone, 6th rib, and suspicious C2 vertebral lesion.
b. Detected multifocal distant skeletal involvement in one scan (because of whole body imaging), including possible metastatic-like lesion, radically impacting staging and prognosis.

### 3.6 Case 6

A 46 year old female, recently diagnosed case of Type 2 Diabetes mellitus and hypothyroidism and hypertension since 3 years, presented with history of left side of face since 25 days, and numbness over jaw and upper alveolus and was diagnosed with mucormycosis. She had no history of fracture, dental extraction, back pain, or any other joint pain. Alkaline phosphatase was found to be raised (206.4 IU/L), and Serum Calcium was within normal range. 99m Tc MDP Bone scintigraphy: (Supplementary Figure d) revealed involvement of left zygomatic region with additional SPECT/CT images confirming involvement of left zygomatic bone, frontal process of left maxilla bone, bony sphenoid, upper alveolus bilaterally (likely to be caries).

Incremental value of 99m Tc MDP Bone scintigraphy:

a. SPECT/CT showed left zygomatic bone, frontal process of left maxilla, sphenoid, and upper alveolus bilaterally (probably caries).
b. Helped differentiate alveolar pathology vs. post-op changes, and revealed deeper spread than expected from clinical or MRI data.

### 3.7 Case 7

A 41 year old male, recently diagnosed with Type 2 Diabetes mellitus, with mucormycosis, underwent recent surgery (right medial maxillectomy and right orbital exenteration) and presented with of post-operative pain over left side of face. CEMRI BRAIN was suggestive of residual disease in the right frontal, ethmoidal, sphenoidal sinusitis with enhancing contents along walls of post-operative right orbit and maxilla. 99m Tc MDP Bone scintigraphy planar (Supplementary Figure e) revealed involvement of nasal, right frontal and maxillary region as well SPECT/CT images revealing lesions at the right frontal bone, right zygomatic bone, lesser wing of sphenoid- right aspect, nasal bone-right aspect, body of sphenoid.

Incremental value of 99m Tc MDP Bone scintigraphy:

a. SPECT/CT revealed lesions in right frontal and zygomatic bone, nasal bone, and lesser wing and body of sphenoid.
b. Gave more complete picture of bony skull base and facial bone involvement, compared to residual sinusitis seen on MRI.

### 3.8 Case 8

A 42 year old male, known Type 2 Diabetes mellitus, and recent endoscopic medial maxillectomy with ethmoidectomy, frontal and sphenoid sinusectomy and right optic nerve decompression/septectomy presented with history of swelling over right eye and face. She had history of trauma to right wrist 1 year back, no history of dental extraction, back pain, or any other joint pain. CEMRI Brain and head was suggestive of invasive fungal sinusitis stage III (suppl data). 99m Tc MDP Bone scintigraphy planar (Supplementary Figure f) involvement in the nasal and right maxillary region, as well inclusion of SPECT/CT images revealed involvement in the nasal bone and inferior part of nasal septum, lesser wing of sphenoid-right aspect, greater wing of sphenoid- right aspects.

Incremental value of 99m Tc MDP Bone scintigraphy:

a. Additional sites of involvement nasal bone and septum, lesser and greater wing of sphenoid (right).
b. Enhanced detection of central skull base disease, particularly in sphenoid, aiding in defining advanced involvement.

## 4 Discussion

Post COVID-19 mucormycosis most commonly involves structures of the head mainly base of skull, paranasal sinuses, facial bones and various surrounding vital structures. It is fulminating in its course and requires immediate surgical debridement which may include exenteration. This makes mucormycosis one of the deadliest COVID-19 sequel (9). Our case series shows how 99m Tc MDP Bone scintigraphy using both planar and SPECT/CT can add incremental value to the diagnostic dilemmas posed by this very life threatening and emergent infection (Table 1).

1. 99m Tc MDP Whole body bone scans are of great diagnostic value in presurgical stage for planning type of surgery in cases of Mucormycosis with skeletal involvement as well as in post op stage for diagnosing recurrence or any other distant bony site of involvement (1, 2, 6, 7).
2. Not only planar bone scintigraphy images which may give an overall idea of involvement of various sites throughout the body but additional SPECT/CT images at the sites of involvement as seen on planar images is helpful in accurately delineating the specific bony sites involved in mucormycosis as well as may display other additional sites which may not be able to be picked up and delineated in the planar images.
3. Early Detection of Osseous Involvement: 99mTc-MDP bone scan is highly sensitive for detecting osteoblastic activity, often identifying bone involvement earlier than CT or MRI. Additional sites were detected by SPECT/CT, which were not clearly delineated on anatomical imaging (7, 8).
4. Extent of Disease Mapping: SPECT/CT allows three-dimensional anatomical localization, providing detailed mapping of the disease extent. This is especially important in complex skull base and facial bones where disease can be multifocal or insidious.
5. Differentiation Between Active and Inactive Disease: In a post-operative setting, it is challenging to distinguish residual/recurrent disease from post-surgical changes on MRI alone. Increased radiotracer uptake on bone scan suggests ongoing osteoblastic activity, indicating active disease rather than mere scar or surgical change.
6. Guiding Biopsy or Surgical Planning: Helps identify the most metabolically active site for biopsy or surgical debridement. Aids in pre-surgical planning by showing disease beyond clinically suspected areas (7).
7. Bone scan with SPECT/CT provided incremental diagnostic value over MRI by revealing more extensive bony involvement, helping to distinguish active disease from post-operative changes, and assisting in clinical decision-making regarding further management of suspected recurrent mucormycosis (7, 8).

**Table 1:** Case-wise incremental findings by 99m Tc MDP bone scintigraphy (planar & SPECT/CT).

Case	Additional findings by SPECT/CT	Clinical impact
1	Frontal bone, nasal septum, sphenoid body & wing	Extended skull base mapping
2	Cribriform plate, bilateral greater wing of sphenoid	Bilateral skull base disease
3	Orbital walls, nasal bone, ethmoid, bilateral sphenoid	More extensive orbital/facial spread
4	Pterygoid plate, mastoid, zygomatic, orbital floor	Involvement of skull base + mastoid
5	Extensive—skull base, zygoma, rib, vertebra	Multifocal + distant skeletal lesions
6	Upper alveolus, sphenoid, zygoma	Clarified bone vs. dental disease
7	Nasal, zygoma, sphenoid (body & wing)	Expanded surgical field
8	Septum, sphenoid wings	Confirmed deep skull base disease

### 4.1 Study limitations

1. Small Sample Size: The study included a relatively limited number of patients, which may reduce the statistical power of the results. A small sample size increases the likelihood of random variations influencing the findings and may prevent the detection of subtle but clinically relevant associations.
2. Single-Center Study: All cases were collected and analyzed from a single institution. This may reflect the specific referral patterns, patient demographics, and imaging protocols unique to that center, thereby limiting the external validity and generalizability of the results to other populations or healthcare settings.
3. Potential for Selection Bias: Since the study population was derived from a single center with specific inclusion criteria, there is a possibility of inherent selection bias. Certain patient subsets might be overrepresented or underrepresented, which could influence the observed outcomes.
4. Limited Generalizability: Owing to the small number of cases and single-center nature of the study, the results should be interpreted with caution. Further multicenter studies with larger sample sizes are required to validate these findings and establish their applicability across diverse clinical settings.

## 5 Conclusion

Mucormycosis is a severe fungal infection caused by Mucorales fungi, mainly Rhizopus species, typically found in soil and decaying matter. It occurs through inhalation, skin inoculation, or ingestion, particularly affecting individuals with weakened immune systems, such as those with diabetes, cancer, organ transplants, on immunosuppressive drugs, or COVID-19. The infection can manifest in several forms, including encephalic, cutaneous, gastrointestinal, pulmonary, and rhino cerebral, with the latter being the most common and deadly. Characterized by rapid tissue necrosis and blood vessel invasion, mucormycosis requires urgent medical attention. Treatment includes aggressive surgical removal of necrotic tissue and medical management to bolster the immune system and address underlying conditions. Early detection and intervention are crucial, especially in the context of COVID 19, to improve patient outcomes. CT and MRI are standard of care modalities for imaging mucormycosis, however we found that bone scintigraphy both planar and SPECT/CT provided incremental information which assisted in early detection of osseous involvement, extent of disease mapping (including distant sites), differentiation between active and inactive disease, guiding for site of biopsy and extent of surgery especially as it involves vital structures of the head (Table 1). Thus, bone scan if included as an imaging modality in the management plan of mucormycosis can prove to play a pivotal role in planning of treatment where early diagnosis and accurate management is necessary. While the present study highlights the potential role of bone scintigraphy in the evaluation of patients with foot and ankle pain, the findings are based on a limited number of cases from a single institution. Therefore, larger, multicenter studies with more diverse patient populations are essential to validate these observations and to establish the definitive diagnostic value and clinical utility of bone scintigraphy in this setting.

## Data Availability

The original contributions presented in the study are included in the article/Supplementary Material, further inquiries can be directed to the corresponding author.
